# Relationship-centered care and diversity, equity, inclusion, and belonging in action

**DOI:** 10.1097/NSG.0000000000000209

**Published:** 2025-05-19

**Authors:** Latonia Clark Chalmers

**Affiliations:** At the University of North Carolina at Chapel Hill School of Nursing, **LaTonia Clark Chalmers** is an Assistant Professor and teaches in the pre-licensure and graduate programs.

**Keywords:** belonging, DEIB, diversity, health equity, nursing education, social justice

## Abstract

Nursing practice focused on health equity, social justice, and DEIB (diversity, equity, inclusion, and belonging) provides a lens through which nurses can support the advancement and achievement of optimal health for everyone. This article discusses the importance and strategies for incorporating DEIB into nursing education.

Professional nursing organizations have been explicit with a call to action for nurses to advance and achieve health equity and social justice for all.^1^-^4^ Health equity is defined as the “fair and just opportunity for all people [diversity, inclusion, and belonging] to achieve their full health potential without variation from personal characteristics, historical oppression, and societal influences [equity and social justice].”^5^ Broadly, social justice is about recognizing, understanding, critiquing, and addressing the wrongs created, perpetuated, and maintained by unethical, unfair, and unjust systems, structures, policies, laws, and societal/organizational/interpersonal beliefs, norms, and practices that have oppressed, marginalized, and excluded people, populations, and social identities from equitable access to opportunities, resources, and environments to achieve health equity, optimal outcomes, and human flourishing.^1^,^4^

When caring for diverse individuals and populations (such as diversity in terms of race, ethnicity, country of origin, religion, socioeconomic status, educational level, gender identity or expression, sexual orientation, age, ability/health status, language, or culture), nurses are ethically bound to engage in fair and just social justice nursing practice characterized by “compassion and respect for the inherent dignity, worth and unique attributes of every person” and allyship actions “consistent with the obligation to promote health and to provide optimal care” (see *Definition of key terms*).^1^

From a nursing ethics perspective, a nursing practice focused on health equity, social justice, and DEIB (diversity, equity, inclusion, and belonging) provides a lens through which nurses can support the advancement and achievement of optimal health for everyone.

However, in nursing education, the approach to teaching health equity and social justice varies widely.^6^ When health equity and social justice are included in nursing education, these are often not woven throughout a program's curricula.^7^ In addition, studies conducted with nurse educators reveal challenges with teaching this content in a substantive way related to personal factors, institutional factors, and curricular opportunities.^8^-^11^

Studies also indicate that this is particularly true for the health equity topic of racism.^8^,^9^,^12^ From a learner perspective, nursing students have expressed the need and desire for health equity and social justice education that better prepares them for nursing practice.^13^

Nurse educators can assist with preparing students for practice by facilitating learning that builds a critical understanding of the individual and structural barriers to and facilitators of health equity (structural and social determinants of health); engaging learners in analyzing and critiquing how biased and prejudiced beliefs (for example, “isms” such as ableism, weight bias or sizeism, and racism) and behaviors are antithetical to health equity and social justice and can disrupt and impede relationship-centered care; and teaching the importance of nurses individually and collectively leveraging their position, power, and privilege to offer relationship-centered care that promotes human dignity and optimal health.

This article describes an innovative nursing ethics and DEIB-informed nursing education strategy for building health equity, social justice, and relationship-centered care knowledge, skills, and attitudes in prelicensure nursing students.

## Relationship-centered care

Nursing is an active and accountable process centered around the health and well-being of the patient, family, community, and population. In these caregiving relationships, nurses are “always accountable for their judgments, decisions, and actions,” and nursing ethics require the nurse to actively and intentionally set aside and eliminate any “bigotry, prejudicial bias, and discrimination,” such as “ism”-based beliefs.^1^ Despite DEIB being politicized, and, in many cases, literally stripped from organizational and institutional frameworks, nurse educators must lean into rather than avoid or abandon DEIB values as these values are integrally woven into the fabric and heart of nursing ethics, a nursing practice that advances social justice and health equity-minded nursing, and relationship-centered care.

**Table:** Definition of key terms

Term	Definition
Allyship	As described by the ANA Code of Ethics for Nurses,1 allyship “is an ethical duty that requires intentional interventions, advocacy, and support to eliminate harmful acts, words, and deeds.” Through allyship, the nurse addresses bias, prejudice, and discrimination in all its forms and, with conscious intention, engages in ethical nursing practice that preserves human dignity and promotes social justice and health equity for all.^1^,^29^,^30^
Position	“Position” refers to the nurse's professional role in caring for a recipient or patient (also known as the individual, family, community, or population).1 From a health equity and social justice perspective, “position” also includes the nurse's social identities. Ethically, nurses speak out against all historical and current systems and forms of identity-based (such as ability, age, class, health status, race, ethnicity, county of origin, sex, gender, and religion) oppression, marginalization, exclusion, dehumanization, discrimination, bias, and prejudice and engage in ethical nursing practice that improves health outcomes for all patients. Ethically, nurses use their professional position and social identities to engage in allyship for all, especially for patients, families, and communities who are marginalized, disenfranchised, underserved, and underresourced. Nurses are ethically bound to use their position individually and collectively to promote social justice and health equity for all.^1^
Power	“Power” refers to the nurse's ability and capacity to act in the best interest of the recipient of care for optimal health outcomes. The ANA Code of Ethics for Nurses1 identifies the nurse as being in an inherent position of power in the nurse-patient relationship, as the patient depends on the nurse for healthcare. Historically and presently in the US, groups of people (based on identity) have lacked power, equitable and fair treatment, access to resources, and human flourishing opportunities due to unjust systemic, societal, and structural barriers. Nurses are ethically bound to use their power individually and collectively to promote social justice and health equity for all.^1^
Privilege	“Privilege” refers to the opportunities, access, and rights nurses have based on having a nursing professional identity. Beyond the nursing professional identity, the nurse may have privileges (such as opportunities, access, rights, and advantages) connected to their respective social identities. Historically and presently, various groups (based on social identity) have had/have “privilege” accessing optimal healthcare over other groups due to societal, structural, and systemic factors. Nurses are ethically bound to use their privilege individually and collectively to promote social justice and health equity for all.^1^

Anchoring relationship-centered care is the action verbs of “relating” and “centering.” Through a health equity, social justice, and positionality-informed consciousness^14^ lens, when relating to and centering the individual/family/community/population, health equity-minded nurses must have the knowledge, skills, attitudes, and competencies^4^ to use their nurse position, nurse power, and nurse privilege to holistically care for the individual, family, community, and population.^1^

Nurses have an ethical obligation to understand the socio-historical-political-cultural contexts of the patient, family, community, and population^1^,^4^,^15^ and use their position, power, and privilege to engage in ethical relationship-centered nursing practice by: (1) offering compassionate, trauma-informed, transpersonal caring to diverse populations^1^,^4^,^16^,^17^; (2) advancing health equity in words, deeds, and actions^1^-^4^,^18^,^19^; (3) creating, fostering, and maintaining inclusive, respectful, and culturally sensitive, culturally responsive, culturally relevant, culturally informed, culturally safe, and culturally concordant healing environments^1^,^4^,^15^,^20^; and (4) prioritizing centering the humanity and dignity of the individual, family, community, or population to nurture a sense of belonging, advocate for health as a human right, and promote human flourishing.^1^

From a nursing education perspective, rather than nurse educators solely lecturing about nursing ethics and what needs to be done to advance health equity, nurse educators must actively support learners to consciously connect nursing ethics and knowledge to real-life contexts and action.^4^,^13^,^15^,^17^,^21^ As described in the literature, health-equity minded, social justice advancing, relationship-centered nursing care can be disrupted by nursing practice influenced and contaminated by isms, biases, and stigma. Building knowledge, skills, and attitudes about addressing the healthcare problems of isms, biases, and stigma was the focus of the series of assignments described in the next sections of this article.

## Project background

In a Foundations of Nursing course with key themes of relationship-centered care, nursing ethics, diversity, and inclusion and taught during the summer semester to prelicensure, second-degree nursing students, student feedback and faculty observations identified the need for more active learning opportunities for critical thinking and problem-solving.

From Summer 2023 to Spring 2024, feedback was gathered informally (through students verbally sharing) and formally (through course evaluations). Students also shared feedback with the former Senior Associate Dean (AD) of Diversity and Inclusion and specifically raised concerns about what students perceived as a lack of content related to racism and health equity in both this course and throughout the undergraduate nursing program.

Parallel to the findings of De Sousa et al., students expressed a desire to be engaged in learning strategies that “go beyond superficial engagement and awareness and provide the *how to* enact social justice in professional practice.”^13^ With the course objectives, AACN *Essentials*, ANA *Code of Ethics*, and student feedback in mind, the author aimed to creatively explore ways to shift from lecture-based teaching (passive learning) to an andragogical approach that emphasizes students being actively and collaboratively engaged in learning, problem-solving, and applying knowledge.^4^,^15^,^22^

Prior to the 2024 summer semester (mid-May to early August), the author and sole coordinator for a class of 87 students conducted a literature review to identify common biases/isms in the healthcare setting and then developed a series of four interconnected assignments entitled “Healthcare Problems & Relationship-Centered Care in Action Series” based on the biases/isms (such as healthcare problems) identified from the literature review.

## Literature review

Keywords used in the literature search included “nurse,” “bias,” “healthcare worker,” “stigma,” “isms,” and specific isms, for example, classism, ageism, and racism. Pertinent to this nursing course, three of the retrieved articles were authored by nurses and published within the last 5 years (see *Literature review articles on bias and stigma [condensed]*). From these three articles, the following biases/healthcare problems were identified and used for the assignment series: ageism, classism, anti-LGBTQIA2S++ (Lesbian, Gay, Bisexual, Transgender, Queer, Intersex, Asexual, Two-Spirit, and inclusive of all identities not represented by the letters)/anti-sexual and gender minorities (SGMs) attitudes, bias/stigma against people with mental health problems, racism, sizeism (weight bias), and bias/stigma against people with substance use disorders (SUD).^23^-^25^ The literature review also identified ableism and classism as commonly occurring in healthcare workers, and these were also included in the assignment series.

**Figure:**
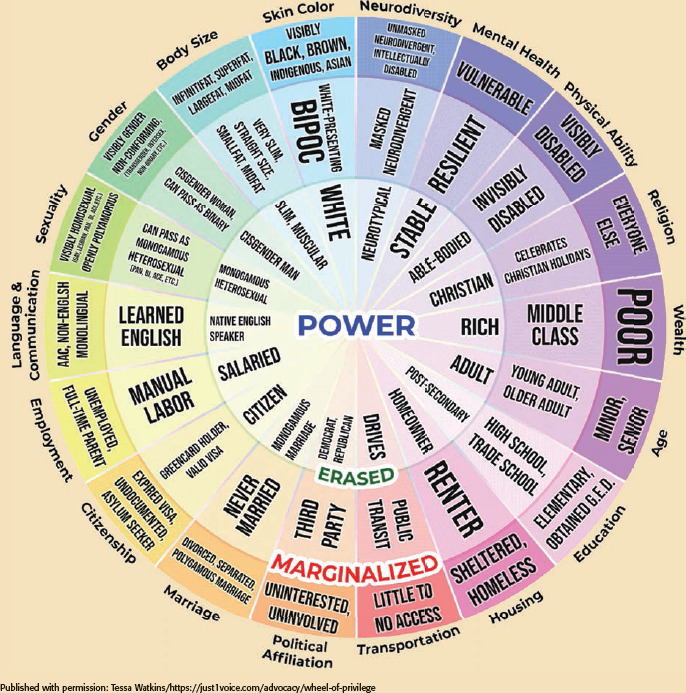
Intersectionality wheel of privilege as observed in the USA

Of note, because of the size of the class, length of the summer session, number of classes, and goal for students to work in small groups with no more than six learners, the number of isms/biases selected was capped at eight.

**Table:** Healthcare problems and relationship-centered care in action series: Assignment 1, Part 2

Deliverable	Components
**Deliverable 1: Slide presentation**	Content (based on the literature) Problem Definition: Clearly defines the healthcare problem Uses at least two reputable sources to support the definition Stereotypes, Stigma, Bias: Identifies common stereotypes, stigma, and biases associated with the problem based on the literature Non-Relationship-Centered Care: Provides examples of care that are not relationship-centered based on literature (e.g., non-therapeutic communication, non-person-centered language) Impact on Health & Delivery: Explains the impact of the problem on health (considering the person/family as a whole) with evidence of harm caused Explains the impact of the problem on healthcare delivery with evidence of harm caused Data & Statistics: Presents relevant data and statistics about the extent of the bias/stigma-based healthcare problem Structural/systemic issues: Clearly identifies structural/systemic issues contributing to the problem
**Deliverable 2: Case study script** (submitted as a Word document)	Content: Person/Family Description: Describes two visible identities (e.g., employment status, age) of the person/family experiencing the problem Describes three invisible identities (e.g., trauma survivor, disability, sexual orientation) of the person/family experiencing the problem Problem Illustration (based on the literature review): Clearly illustrates the assigned healthcare problem in action (i.e., ineffective approaches to care that convey the isms/biases/stereotypes associated with the healthcare problems, are not trauma-informed, and do not center the needs/goals of the patient/family) Includes examples of verbal and non-verbal communication that demonstrate non-therapeutic care (e.g., non-therapeutic body language, non-therapeutic verbal communication, language that is not person-centered, language that is complicated/wordy and not following health literacy principles) Case study is mindful of potential triggers and avoids causing harm to students with lived experiences
**Deliverable 3: Case study video** (3-5 minutes; submitted as a link, MP4, or other easily accessible video format. Permission needed for longer video)	Relationship-Centered Care: Video clearly demonstrates relationship-centered care in action that is culturally relevant and responsive for the specific community/population depicted in Deliverable 2 (community/population impacted by the assigned healthcare problem) as evidenced by: Communication & Building Trust & Rapport: Video clearly demonstrates healthcare worker(s) effectively building trust and rapport with the patient/family through verbal and non-verbal communication that demonstrates compassion, respect, and regard for the person/family's humanity and dignity Cultural humility & Power Dynamics: Video clearly demonstrates cultural humility and awareness of the healthcare worker's position, power, and privilege in relation to the person/family. The video addresses potential healthcare disparities or inequities faced by the community represented in the case study Person-Centered Approach: Video clearly utilizes person-first, inclusive language and demonstrates a focus on the patient's/family's needs and goals Communication & Health Literacy: Video clearly incorporates therapeutic communication (verbal and non-verbal) and health literacy principles to ensure clear understanding for the patient/family

## Assignment development

A total of four assignments were created based on the literature review results. Two assignments were team/group-based (Assignments 1 and 3), and two were individually completed (Assignments 2 and 4).

Due to the sensitive topics covered by the new assignment series and for author accountability and transparency, faculty peer feedback was requested and received on the assignments. The assignments were also rigorously reviewed by the Senior Associate Dean for Inclusive Excellence, updated as needed, and finally received approval from the Senior Associate Dean.

The identified isms/biases (i.e., healthcare problems) were posted under the respective assignment heading in the course's online learning management system (LMS). Although one of the goals of the series was for students to be able to recognize and ethically respond to all the isms/biases covered by the series of assignments, there was a brief window of time when students could self-select or self-opt out of the healthcare problem to which they preferred to be assigned/did not want to be assigned. With psychological safety in mind, this trauma-informed classroom option for self-selection was provided to offer space for students to volunteer for a healthcare problem they are passionate about or avoid a healthcare problem that might be triggering or upsetting.^26^,^27^ For example, a student with lived experience with a SUD via their own history with a SUD or a family member's or friend's SUD may or may not have wanted to be assigned to the bias against people with SUDs group. Most students declined the option to self-select/opt-out and were assigned to a group by the author.

For each of the eight healthcare problems, two small groups consisting of the same number of students (five to six students) were assigned (for example, Ageism Group A and Ageism Group B each had six students). Four of the eight healthcare problems were assigned eight groups of six students, and four of the healthcare problems were assigned eight groups of five students. To assist with completing the assignments, the author selected peer-reviewed articles and other resources about each healthcare problem and posted these to the LMS. Student groups were also encouraged to search the literature and identify and use scholarly references to complete the assignments.

The four assignments were designed to facilitate students actively applying course content (i.e., relationship-centered care in action). During class and leading up to the assignments, topics covered in class included health equity and social justice; whole health/holistic nursing; transpersonal caring; person/relationship-centered care; biases, stigma, and stereotypes; cultural humility and cultural safety; person first, inclusive language; therapeutic communication; trauma-informed care; spiritual care; intersectionality, positionality, power, and privilege (*see Intersectionality wheel of privilege as observed in the USA*; structural determinants of health; allyship; racism; nursing history, theory, and ethics; and health literacy and the use of plain language in nursing practice.

**Table:** Healthcare problems and relationship-centered care in action series: Assignment 4 - Personal reflection

Completed by each student individually. Student choice: You may complete this personal reflection via submission of a video, paper, a VoiceThread (with captions), a slideshow, a poem, or some other artistic expression as long as there is a response to each of the required elements. If you are submitting anything other than a written deliverable document (i.e., text document, slideshow), you are required to include an outline (formatted as a Word document) detailing your audio/visual presentation.	
**Component**	**Description**
**Introduction**	Introduce yourself and include a description of at least one of your visible identities and three invisible identities that you feel comfortable sharing. Identify the name of your assigned healthcare problem (the bias/stigma problem that you have been assigned to analyze during this semester).
**Group experience**	Assignment 1's group experience: Identification of group members' contributions/roles What was your experience like working in this group? Thoughts about your group's Assignment 3 presentation
**Personal introspection**	As a student nurse/nurse, it is possible for you to have a physical, mental, emotional, and/or spiritual response to observing/experiencing healthcare problems and responding to healthcare problems. Describe your physical, mental, emotional, and/or spiritual experiences creating and responding to your assigned healthcare problem. Please describe the domains that are relevant for you (i.e., the description of your lived experiences could include one domain, two domains, three domains, or all four) Regarding your assigned healthcare problem, what are at least two allyship (relationship-centered care in action) pearls that you are taking away from this assignment?
**Relationship-centered care in action: Continuing the journey of social justice nursing (For students assigned to racism, alternate instructions have been provided)**	Given the pervasiveness of race-based discrimination (many healthcare organizations and health scholars have identified racism as a public health crisis) that negatively impacts health and healthcare delivery in the healthcare setting, what is your social justice commitment to addressing racism in practice, that is, how will you use your position, power, and privilege as a nurse to advance health equity and address racism in the healthcare setting and beyond? Choose two other healthcare problems/cases that were presented (different than your assigned problem in healthcare) and describe two allyship pearls for each of the two healthcare problems/cases. How will you use your position, power, and privilege as a nurse to advance health equity for the populations directly impacted by each of the healthcare problems that you have selected? What is your social justice commitment to addressing your assigned healthcare problem in practice, that is, how will you use your position, power, and privilege as a nurse to advance health equity for the populations impacted by your assigned healthcare problem?
**Closing thoughts/conclusion**	Please share your closing/concluding thoughts about the Healthcare problems and relationship-centered care in action assignment series. In your closing/conclusion, share any professional and personal growth you have experienced during this series of assignments.

Prior to the first day of class, the required reading included the two nurse-written articles “Words matter: Labelling, bias, and stigma in nursing” by Dr. Anna Valdez^23^ and “The State of the Science of Nurses' Implicit Bias: A Call to Go Beyond the Face of the Other and Revisit the Ethics of Belonging and Power” by Dr. Holly Wei and team.^24^ Valdez's very transparent and relatable article provided students with a nurse perspective, humanized and normalized the nurse having biases, humanized the patient on the receiving end of the biases, and provided students with an opportunity to be open and honest about their own lived or witnessed experiences with biases and nonperson human-centered language.^23^ With the article by Wei et al.,^24^ students received a closer look at the breadth, depth, and impact of implicit biases in nursing and were provided a better understanding of the rationale for the series of assignments. The use of these two articles was one of the multiple strategies used during the course and assignment series to support psychological safety.

Assignment 1 focused on students working together to create four literature-informed deliverables about the assigned ism/bias-based healthcare problem. For Deliverable 1, students submitted a literature-informed outline with required elements for faculty review, feedback, and approval. Deliverable 2 was a detailed, literature-informed case study script of the healthcare problem (for example, an “ism”) in action. Deliverable 3 was a slideshow that included the definition of the problem, its impact on health and healthcare, relevant statistics, and structural or system contributors to the problem. Deliverable 4 was a video demonstrating a relationship-centered, health-equity-minded remedy to the scenario detailed in Deliverable 2 (see *Healthcare problems and relationship-centered care in action series: Assignment 1, Part 2*).

For Assignment 2, students individually analyzed and responded to the case study of the corresponding group (for example, if the student was assigned to Ageism Group A, then they responded to Ageism Group B's case study). With psychological safety in mind, all case studies were reviewed and edited as needed prior to being released for Assignment 2 (see *Healthcare problems and relationship-centered care in action series: Assignment 2 components*).

Assignment 3 was a class presentation, and each group presented their slides (from Assignment 1) in class, shared their group-created video, and co-led the class with faculty.

For Assignment 4, students were given a prompt and submitted a reflection synthesizing the learning from the four assignments. Assignment 4 also invited students to share their thoughts, feelings, and feedback about the assignment series (see *Healthcare problems and relationship-centered care in action series: Assignment 4 - Personal reflection*).

## Student feedback

Despite the multiple moving parts to this assignment series, verbal and written feedback received from students was overwhelmingly positive. Feedback about the assignment was collected from all the students during Assignment 4, and some students opted to provide feedback via the end-of-course evaluation. During the class presentations and since the end of class early August 2024, about 25% of the class shared direct verbal feedback to the author. During the class presentations, students conveyed a sense of great pride in being active in their learning and sharing it with their peers. Some students shared that they had learned more by “doing” the “how to” of the course content than they would have by being “lectured to.” Many students seemed surprised to learn that they held ageist, ableist, and classist beliefs. Some were willing to acknowledge long-standing biases (such as weight bias and biases against people with mental health challenges and SUDs) and the need to challenge these biases. Overall, students acknowledged a deepening of understanding of the suffering of those impacted by the “isms” and expressed newfound compassion and empathy. All students identified allyship pearls and made social justice commitments to responding to health and healthcare inequities in practice.

## Opportunities and recommendations

There are several limitations to this innovative approach to teaching about health equity, nursing ethics, and social justice-minded nursing. No validated tools were used to measure student attitudes or learning outcomes. In addition, this was the first iteration and delivery of this assignment series, and opportunities for clarifying assignment expectations were discovered during the series. Other “isms” and biases could not be included because of time constraints. On the other hand, a single ism could have been the focus of the entire semester. Students also expressed the desire to have more class days for in-class presentations to facilitate more in-depth class and small group breakout discussions.

Although the hope is that the learning that took place during the assignments impacts the nursing practice of participants, the actual short- and long-term impacts are unknown. As described by Graefe et al.,^7^ students would benefit from the nursing program adopting a more cohesive and integrative approach to teaching health equity and social justice-minded nursing. At the author's institution, current interprofessional efforts are underway to examine how to better incorporate programmatic or cross-departmental health equity curricula.

Future recommendations include experientially engaging students with community members or simulated patients impacted by the isms/bias-based healthcare problems and including more related case studies. Finally, there are opportunities to collaborate with the Associate Dean of Teaching, Learning, and Innovation to streamline the assignments and use creative tools to embed more interactive learning within the LMS.

## Final considerations for nurse educators

This author fully acknowledges her power, position, and privilege to teach this content and understands that the work of health equity and social justice is collective, requires lifelong learning, and can be exhausting. The work is even more exhausting when other nurse educators and nurses have not done their work—internally and externally. Regardless of an individual's belief about DEIB, nurses have an ethical and moral obligation and responsibility to center, prioritize, and advance the health, healing, recovery, and humanity of all people. As stated by Garland and Batty, “prior to selecting teaching and learning strategies, every nurse educator must reflect on their position within the world and deeply consider the inherent assumptions that may perpetuate oppression and imperceptible discrimination.”^28^ Just as nursing students are expected to engage in reflective and reflexive practice, nurse educators must do the same: embrace areas for growth and lean into health equity and social justice-minded nursing education.

## Literature review articles on bias and stigma (condensed)

- Valdez A. Words matter: labelling, bias and stigma in nursing. *J Adv Nurs*. 2021;77(11):e33-e35. doi:10.1111/jan.14967.
- Wei H, Price Z, Evans K, Haberstroh A, Hines-Martin V, Harrington CC. The state of the science of nurses' implicit bias: A call to go beyond the face of the other and revisit the ethics of belonging and power. *ANS Adv Nurs Sci*. 2023;46(2):121-136. doi:10.1097/ANS.0000000000000470.
- Wolf L, Delao A, Perhats C, et al. The experiences of United States emergency nurses related to witnessed and experienced bias: A mixed-methods study. J *Emerg Nurs*. 2023;49(2):175-197. doi:10.1016/j.jen.2022.11.008.
- Appelgren M, Bahtsevani C, Persson K, Borglin G. Nurses' experiences of caring for patients with intellectual developmental disorders: A systematic review using a meta-ethnographic approach. *BMC Nurs*. 2018;17:51. doi:10.1186/s12912-018-0316-9.
- Christakis DA, Iezzoni LI. Calling on the USPSTF to address ableism and structural ableism. *JAMA*. 2023;330(14):1327-1328. doi:10.1001/jama.2023.17092.
- Derbyshire DW, Keay T. “But what do you really think?” Nurses' contrasting explicit and implicit attitudes towards people with disabilities using the implicit association test. *J Clin Nurs*. 2024;33(11):4342-4353. doi:10.1111/jocn.17097.
- Lundberg DJ, Chen JA. Structural ableism in public health and healthcare: A definition and conceptual framework. *Lancet Reg Health Am*. 2023;30:100650. doi:10.1016/j.lana.2023.100650.
- VanPuymbrouck L, Friedman C, Feldner H. Explicit and implicit disability attitudes of healthcare providers. *Rehabil Psychol*. 2020;65(2):101-112. doi:10.1037/rep0000317.
- Gonzalez D, Kenney GM, O'Brien C, McDaniel M, Karpman M. Publicly insured and uninsured patients are more likely than other patients to be treated unfairly in health care settings because of their coverage type. Robert Wood Johnson Foundation. 2022. www.rwjf.org/en/insights/our-research/2022/09/publicly-insured-and-uninsured-patients-are-more-likely-than-other-patients-to-be-treated-unfairly-in-healthcare-settings-because-of-their-coverage-type.html. Accessed May 24, 2024.
- Hilliard E, Twiss B, Pearson M. Upward bound students' experience with bias in healthcare: An application of critical race theory. *Nurs Open*. 2023;10(8):5682-5692. doi:10.1002/nop2.1814.
- Mahabir DF, O'Campo P, Lofters A, Shankardass K, Salmon C, Muntaner C. Classism and everyday racism as experienced by racialized health care users: A concept mapping study. *Int J Health Serv*. 2021;51(3):350-363. doi:10.1177/00207314211014782.
- Meidert U, Dönnges G, Bucher T, Wieber F, Gerber-Grote A. Unconscious bias among health professionals: a scoping review. *Int J Environ Res Public Health*. 2023;20(16):6569. doi:10.3390/ijerph2016656.

## Healthcare problems and relationship-centered care in action series: Assignment 2 components

**Critical consideration of uncaring practices**

- The vignette analysis clearly identifies at least three ways in which caring and compassion were not demonstrated.
  - Examples link to specific concepts, such as biases, stereotypes, non-therapeutic communication.
  - Explanation demonstrates understanding of how these practices negatively impact healthcare experiences.
  - Relationship-centered care approach
- **A relevant nursing theory is identified and explained**.
  - A connection between the theory and relationship-centered care is established.
  - At least two relevant nursing ethics principles are identified and explained.
  - Evidence-based approaches to relationship-centered care are described in detail.
  - The description demonstrates how care would be person/family/community-centered.
- **Application to the vignette**
  - The student explains how they would provide relationship-centered care in the specific scenario.
  - The explanation demonstrates cultural humility and a holistic approach to care.
